# Function and control of human invasive trophoblast subtypes: Intrinsic vs. maternal control

**DOI:** 10.1080/19336918.2015.1089376

**Published:** 2015-09-29

**Authors:** Philipp Velicky, Martin Knöfler, Jürgen Pollheimer

**Affiliations:** Department of Obstetrics and Fetal-Maternal Medicine, Reproductive Biology Unit, Medical University of Vienna, Vienna, Austria

**Keywords:** decidua, EVT, invasion, placenta, preeclampsia, trophoblast

## Abstract

The establishment of a functional placenta is pivotal for normal fetal development and the maintenance of pregnancy. In the course of early placentation, trophoblast precursors differentiate into highly invasive trophoblast subtypes. These cells, referred to as extravillous trophoblasts (EVTs), penetrate the maternal uterus reaching as far as the inner third of the myometrium. One of the most fundamental functions of EVTs is the transformation of spiral arteries to establish the uteroplacental blood circulation assuring an adequate nutrient and gas supply to the developing fetus. To achieve this, specialized EVT subpopulations interact with maternal immune cells, provoke elastolysis in the arterial wall and replace the endothelial cells lining the spiral arteries to induce intraluminal vascular remodeling. These and other trophoblast-mediated processes are tightly controlled by paracrine signals from the maternal decidua and furthermore underlie an intrinsic cell-type specific program. Various severe pregnancy complications such as preeclampsia or intrauterine growth retardation are associated with abnormal EVT function, shallow invasion, and decreased blood flow to the placenta. Hence a better understanding of human trophoblast invasion seems mandatory to improve therapeutic intervention. This approach, however, requires a profound knowledge of the human placenta, its various trophoblast subtypes and in particular a better understanding of the regulatory network that controls the invasive phenotype of EVTs.

## Introduction

The objective of this article is to review and discuss our current understanding of regulatory mechanisms that guide and control human trophoblast invasion. Consequently, processes underlying placental formation and those involved in the function of non-invasive trophoblast subpopulations including villous cytotrophoblasts (vCTBs) and syncytiotrophoblasts (STs) will only be discussed in brief. Accordingly, we will firstly provide a short overview on placental development in humans and then focus on discussing formation and function of different human invasive trophoblast subtypes and the regulatory network that controls these cells.

**Figure 1.**
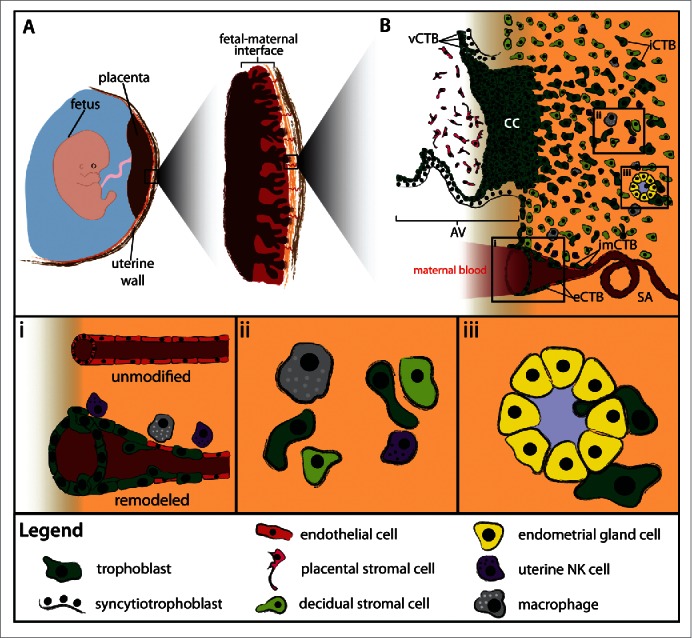
Human trophoblast invasion. (A) The placenta connects the fetus to the uterine wall and establishes a vascular connection between mother and child. The placenta is structured as villous tree and is in direct contact with maternal blood and, thus referred to as hemochorial. The site where the placenta comes in direct contact with the maternal decidua is called the fetal-maternal interface. (B) During early pregnancy, vCTBs fuse to form multinucleated STs, which surround the placental villus. STs transport nutrients and gases from the maternal to the fetal circulation and represent the major endocrine unit of the placenta by secreting hormones such as chorionic gonadotropin, placental growth hormone or placental lactogen. AV form cell columns that attach to the maternal decidua and give rise to the EVT lineage. Invasive EVTs can be divided into iCTBs, which invade the decidual stroma and become terminally differentiated multinucleated GCs, or eCTBs. (i) The latter colonise the lumen of uterine spiral arteries and together with iCTBs, macrophages and uNK cells convert these vessels into larger conduits to guarantee adequate blood flow to the growing fetus. (ii) Various decidual cell types such as macrophages, uNK cells and decidual stromal cells interact with the interstitial cytotrophoblasts in order to control iCTB invasion in an in a spatio-temporal manner. (iii) In addition, iCTBs invade and replace the epithelium of endometrial glands. AV, anchoring villus; CC, cell column; EVT, extravillous cytotrophoblast; eCTB, endovascular cytotrophoblast; iCTB, interstitial cytotrophoblast; SA, spiral artery; ST, syncytiotrophoblast; uNK, uterine natural killer cell.

The first cell differentiation event in mammalian development occurs during the transition from the morula to the blastocyst and leads to the establishment of 2 distinct cell lineages: the trophectoderm (TE) and the inner cell mass (ICM). While the latter population gives rise to the embryo, the TE is the origin of trophoblasts, which make up the epithelial compartment of the placenta. According to its basic function, which is to control fetal maternal exchange, the placenta is the prerequisite for the survival of the mammalian embryo. Remarkably, placental structure and diversity of trophoblast subtypes differs greatly among all mammals. Most rodents and primates, including humans, develop a so-called hemochorial placenta, being defined by direct contact between maternal blood and trophoblasts (Fig. 1A). Another feature of hemochorial placentas is their highly invasive placentation reaching deep into the pregnant uterus, referred to as decidua. In humans decidualization is induced by rising progesterone and cyclic AMP levels during the secretory phase of the menstrual cycle and is characterized by extensive remodeling events in the endometrial compartment. These changes include the transformation of spindle-shaped endometrial fibroblasts to large polygonal decidual stromal cells (DSCs) and the secretion of high amounts of insulin-like growth factor 1 and prolactin.^1^ Vasculogenesis and angiogenesis occur during the proliferative and secretory phase inducing vessel elongation and coiling as well as growth of spiral arteries, respectively.^2^ Another hallmark of decidualization is the expansion of a unique immune cell population, so-called uterine natural killer (uNK) cells (see below).^3-5^ Whether uNK cells derive from peripheral tissue sources.^6^ or from tissue-resident progenitors.^7^ is still a matter of debate. Human trophoblasts invade the decidua as deep as the inner third of the myometrium and therefore exhibit the most pronounced infiltration of the fetal maternal interface among all eutherian mammals.^8^ Deep placentation during human pregnancy most likely owes to the exceptionally rapid growth of the fetal brain demanding intensive support with nutrients.^9^ Trophoblast invasion of the decidua is necessary to establish pivotal changes in the fetal-maternal environment such as anchorage of the placenta to the decidua, remodeling of the vasculature (see below) as well as cross-talk to maternal decidual stromal cells and lymphocytes, including uNK cells.^10,11^ Failures in placentation and function of trophoblasts during early stages of gestation are associated with numerous pregnancy diseases such as miscarriage, preeclampsia (PE) and intrauterine growth restriction (IUGR).^12,13^ Indeed, uterine vascularization is already impaired in the first trimester of pregnancy in women who later on develop PE.^14-16^ PE and IUGR affect approximately 5-8% of pregnancies and may cause severe conditions during pregnancy, including hypertension and proteinuria in the mother as well as decreased fetal growth and may even lead to maternal or fetal death.^17,18^

In humans, trophoblast-mediated invasion of the decidua is accomplished by a specialized subtype referred to as the extravillous trophoblast (EVT). EVTs arise from a single epithelial cell layer consisting of so-called vCTBs. These cells reside at the basement membrane of placental villi, which separates trophoblasts from the underlying mesenchymal villous core. vCTBs provide a population of progenitor cells that are able to differentiate into 2 distinct entities, the ST and EVT lineage. STs form a continuous multinucleated syncytium that is in direct contact with maternal blood within the intervillous space. These terminally differentiated trophoblasts secrete numerous pregnancy-maintaining hormones including chorionic gonadotropin (hCG), human placental lactogen (hPL), estrogen and progesterone into the maternal circulation.^19^

## Invasive Trophoblast Subtypes and Their Function

EVT formation occurs at sites where placental villi attach to the decidua. These types of villi are named anchoring villi and form so-called cell columns. These consist of highly proliferative proximal trophoblasts, which differentiate into non-dividing trophoblasts at the distal end of the cell column (Fig. 1B). This differential switch is characterized by the upregulation of EVT-specific marker genes including HLA-G,^20^ integrin α (ITGA) 1 and -5,^21^ T-cell factor 4 (TCF4).^22^ as well as a disintegrin and metalloproteinase domain (ADAM) 12.^23,24^ Upon contacting the decidua, distal cell column trophoblasts start to either infiltrate the decidua as interstitial cytotrophoblasts (iCTBs) or colonize spiral arteries, referred to as endovascular cytotrophoblasts (eCTBs).^25^ Remarkably, during the first weeks of gestation eCTBs plug spiral arteries, and later on in pregnancy they transform these vessels into larger conduits in order to deliver low-pressure, high blood flow to the growing fetus.^11^ Trophoblast-mediated occlusion of the arterial openings into the intervillous space is believed to prevent precocious onset of the maternal-placental circulation, which possibly results in oxidative damage of the fetal-placental unit due to the generation of reactive oxygen species.^26,27^ Indeed, disorganized early onset of blood flow and incomplete plugging of the maternal vessels are features of miscarried pregnancies.^28,29^ As mentioned above, by the end of the first trimester of pregnancy (10th – 12th week) trophoblast plugs disappear from the spiral arteries and eCTBs replace the vascular endothelium to induce intraluminal remodeling of the spiral arteries.^11^ This event facilitates a vascular connection between mother and fetus and marks the transition from a histotrophic toward hemotrophic nutrition of the conceptus. Moreover, it is generally believed that EVTs do not invade uterine veins since ephrin B1 expressed on trophoblasts and EPHB4, a receptor associated with venous identity, generate a repulsive signal.^30^ However, it appears that this hypothesis certainly deserves further attention since some data exist to suggest interaction between uterine veins and trophoblasts in humans and macaques.^31,32^ iCTB invade the decidua as deep as the first third of the myometrium, where these cells undergo a final differentiation step into multinucleated trophoblasts giant cells.^8^ It is believed that their formation likely provides a mechanism which prevents deeper penetration into the uterine wall. In addition, these cells produce pregnancy-specific hormones such as hPL and hCG.^33^ It is still a matter of debate whether giant cells are formed by cellular fusion, aggregation or failed cytokinesis. Since iCTBs tend to form trophoblastic clusters at distal parts of the decidua it may very well be that giant cells are indeed formed by trophoblast aggregation as recently suggested.^33^ iCTBs also engage in the remodeling process of spiral arteries provoking the transformation of narrow vessels with relatively high resistance into highly dilated, low-resistance conduits. Transformation of the decidual and myometrial spiral arteries involves the interplay of the placenta-derived iCTB as well as maternal uNK cells and macrophages.^34^ Recent data suggest that macrophages and uNK cells initiate remodeling of spiral arteries in order to disrupt the tightly packed vascular layer of smooth muscle cells and extracellular matrix (ECM).^35^ In particular uNKs cells were found to secrete an array of factors including angiotensin 1 and 2, vascular growth factor c and metalloproteinases (MMP) promoting ECM degradation and vSMC disorganization.^36^ In a second wave, iCTBs cause further disruption for instance by the secretion of MMP-12,^37^ a functional elastolytic protease or by the induction of apoptosis in endothelial as well as smooth muscle cells.^38-40^ However, whether macrophages and/or uNK are imperative for spiral artery remodeling in humans is currently not known. Previous work in mice shows that depletion of uNK cells results in impaired vascular remodeling rather than loss of pregnancy or failure in implantation.^41^ The absence of uNK cells in rats delays but does not prevent conversion of spiral arteries. The authors of this study concluded that uNK cells induce angiogenesis and disruption of the arterial smooth muscle layer, thereby controlling uterine oxygen tension and invasive trophoblast differentiation.^42^ Different to macrophages and uNK cells, iCTBs reside in the arterial wall hence referred to as intramural CTBs (imCTB). Whether imCTBs can breach the vascular basal membrane, convert into eCTBs and replace endothelial cells is not known. The observation that vascular colonization by eCTBs also takes place at sites where no decidual EVTs are detectable speaks against a contribution of imCTBs.^11^ On the other hand, *in vitro* studies demonstrate that trophoblasts are capable of perfusing into the lumen of unmodified spiral arteries, obtained from uterine biopsies.^43^ Another important feature of iCTBs is their ability to interact with immune cells of the fetal-maternal interface. While non-invasive trophoblast subtypes do not express any pattern of human leukocyte antigen (HLA) class I or II molecules, iCTBs show cell surface expression of HLA-E, HLA-G and HLA-C.^44^ The latter was recently shown to interact with KIR receptors expressed by uNK cells, demonstrating that allo-recognition does occur in the fetal-maternal interface. This interaction is suggested to be critical for reproductive success, since mismatch of HLA-C/KIR haplotypes confers a significantly higher risk for preeclampsia and recurrent abortions.^45^ Finally, trophoblasts have also been noticed to invade endometrial glands.^46^ In general, endometrial glands are supposed to play an important role during early pregnancy but represents hitherto a neglected topic in the field of human reproduction. In other eutherian mammals, for instance in the sheep or horse, endometrial gland-derived proteins, lipids and carbohydrates are well characterized as important sources for the histotrophic nutrition of the embryo.^47^ Indeed, disruption of endometrial gland development in sheep results in faulty implantation and embryonic development, and finally loss of pregnancy.^48^ A similar important role for endometrial-derived factors during early human pregnancy is likely since endometrial glands have been shown to discharge into the intervillous space.^49^ Interestingly, it has been recognized that the endometrial secretions during early pregnancy lack sialic acid endcaps. As a result, endometrial gland-derived factors can only be active in the fetal-maternal interface as any of these factors will immediately be removed from the maternal circulation by asiaglycoprotein receptors in the liver.^50^ At least 2 trophoblast subtypes, the pre-syncytitrophoblast and the iCTBs, may likely coordinate erosion of endometrial glands, which results in the release of glandular secretions into the intervillous space. During early placental development the chorionic sac is surrounded by syncytiotrophoblasts and as this cell layer expands, it erodes the epithelium of adjacent uterine glands.^51^ Interestingly, iCTB were also found to actively invade the glandular epithelium and occasionally replace glandular epithelial cells. iCTB-mediated invasion of EGs may therefore represent an alternative mechanisms, which may support histotrophic nutrition of the embryo prior to the onset of maternal blood flow. Besides provision of nutrients to the embryo, endometrial gland function may also contribute to placental development and implantation by the secretion of specific growth factors including epidermal growth factor (EGF) and endocrine gland-derived vascular endothelial growth factor.^51,52^

## Insights Into the Control of Trophoblast Invasion

In this chapter we shall discuss observational evidences that have been created to unravel the impact of cell-intrinsic regulation of trophoblast invasion and networks that are established between fetal trophoblasts and decidual tissue resident cells. Signaling pathways and transcription factors that lie downstream of this regulatory network have been reviewed extensively^53-55^ and thus will not be discussed.

As pointed out above, establishment of the invasive EVT lineage is preceded by the formation of placental cell columns, which proliferate at their proximal ends and, upon expansion, trophoblasts differentiate, acquire an invasive phenotype and invade the decidua. Due to trophoblast-mediated plugging of the uterine vasculature (see above), early placental development occurs in a hypoxic environment, coinciding with a peak in trophoblast proliferation and placental growth.^56^ This has led to the assumption that hypoxia could be the main trigger of early trophoblast growth and by the end of the first trimester of pregnancy when blood flow is established, normoxia may trigger trophoblast invasion. Indeed, first trimester placental explant cultures cultivated under low oxygen conditions show elevated proliferative activity in trophoblasts.^57,58^ Moreover, it has further been suggested that low oxygen tension-induced proliferation suppresses EVT differentiation and therefore is a negative regulator of trophoblast invasion. The underlying mechanisms, in support of this model, involve hypoxia-inducible factor 1 α-induced expression of tumor growth factor β 3 (TGFβ3), an inhibitor of trophoblast motility *in vitro*.^59^ However, there are various observations and experimental data that speak against this hypothesis. Firstly, this model would suggest that overactivation of the cell cycle in trophoblasts inhibits differentiation and as a consequence invasion. On the contrary, experimental data suggest that trophoblast proliferation is a prerequisite for differentiation. For instance, EGF a potent inducer of trophoblast proliferation also enhances EVT formation and invasion,^60^ although EVTs are not responsive to EGF as these cells do not express its exclusive receptor EGFR.^61^ This suggests that expansion of proliferative trophoblast subtypes will also positively affect the conversion into non-dividing, differentiated EVTs. In support of these findings, hyperproliferative complete hydatidiform mole placentas also show accelerated trophoblast differentiation and invasion.^22^ Finally, experimental hypoxia in the rat was reported to enhance trophoblast invasion and vascular remodeling.^62^ In conclusion, although it is likely that hypoxia is a driver of trophoblast proliferation, existing data to support the notion that normoxia is a trigger of trophoblast invasion and suppressor of EVT differentiation remain controversial.

Various secreted molecules, such as growth factors, cytokines, and chemokines, which have been extensively reviewed,^53,54^ are thought to regulate EVT function in an auto- and/or paracrine manner. However, it is unclear to which extent this regulatory network is dependent on an intrinsic EVT-specific program or on signals originating from maternal cells in the decidua. There are various observations that support the notion that the EVTs ability to invade foreign tissue underlies an intrinsic program or at least does not depend on decidua-specific signals. Firstly, isolated trophoblasts, usually containing undifferentiated cytotrophoblasts as well as EVTs, spontaneously invade artificial matrices including fibronectin and Matrigel.^22,63^ Along these lines, it has been shown that at least a subset of these cells undergo differentiation toward the EVT lineage *in vitro*.^64^ Primary trophoblast cultures induce expression of EVT-specific gene signatures when cultivated on extracellular matrices *in vitro* as demonstrated by the induction of certain integrin signatures, HLA-G or ADAM-12 and downregulation of genes that are specifically expressed by non-invasive trophoblast subtypes such as epidermal growth factor receptor and ITGA6.^23,63,64^ Besides the proposed intrinsic, pro-invasive program in trophoblasts it may very well be that extracellular matrices used in these assays also affect trophoblast behavior. Interestingly, recent data suggest that isolated HLA-G^+^ trophoblasts undergo a further differentiation step when cultivated on fibronectin.^65^ Since fibronectin is expressed and secreted by DSC it may be that decidual contact further enhances differentiation into an invasive phenotype. Interestingly, in contrast to dermal stromal cells, DSCs express serine/arginine-rich protein serine/arginine-rich splicing factor 1 (SRSF1), which leads to more efficient fibronectin bundle deposition into the ECM. The same authors confirm that SRSF1-dependent splicing of fibronectin facilitates trophoblast invasion.^66^ However, fibronectin is also strongly produced by the EVT itself pointing toward auto –and paracrine mechanisms facilitating fibronectin-driven trophoblast differentiation and/or invasion.^67^ As pointed out above, isolated trophoblasts are also able to penetrate unmodified arteries from myometrial biopsies.^43^ In addition, EVT differentiation is unaffected in ectopic pregnancies such as tubal pregnancy, despite the absence of a decidua.^68^ Lastly, transplanted human villous placental explants invade tissue and vasculature of immuno-deficient SCID mice lacking supportive paracrine pregnancy-specific signals.^38^

However, there are various evidences in support of a pivotal decidua-specific role in the regulation of trophoblast invasion. For instance, decidua formation is specific to haemochorial placentation and does not occur in non-invasive epithelia chorial placentas.^10^ In support of this observation, comparative studies in primates show a positive correlation between the degree of decidualization and depth of trophoblast invasion.^69^ Along these lines, it has been suggested that the decidua plays an important role in the provision of restraining signals, which restrict the invasive EVT in spatiotemporal manner. Lessons from placental pathologies, which display no or defective decidua formation, such as placenta creta or ectopic pregnancies support this theory as these disorders are associated with excessive trophoblast invasion.^70^ Examples for such anti-invasive cues include DSC-mediated generation of collagen XVIII-derived endostatin or secretion of TGF-β3, both of which are potent inhibitors of trophoblast motility *in vitro*.^63,71^ Interestingly, both factors were found to be elevated in preeclamptic patients.^72,73^ On the other hand, by critically reviewing existing literature it becomes obvious that decidual cells, including uNK cells, macrophages and DSCs not only provide anti-invasive signals but also a myriad of factors that support EVT motility.^53^ For instance, uNK cells, in contrast to peripheral NK populations, were identified as potent secretors of an array of chemokines, cytokines and growth factors, which induced trophoblast motility both *in vivo* and *in vitro*.^74^ It therefore seems apparent that decidual cells may rather represent a “gatekeeper” function allowing trophoblasts to access maternal tissues and blood supply by achieving an accurate balance between under- and over-invasion. Although the causes of PE remain poorly understood und thus are under intense investigation, impaired trophoblast differentiation, survival and in particular invasion have been attributed to this severe pregnancy-related pathology. In support of this concept, both iCTBs and imCTBs from PE patients were found to be reduced in number,^75-78^ showed signs of increased apoptosis^75^ and were more susceptible to cell death when cultivated *in vitro*.^79^ It the light of this assumption it is interesting to note, that specific non-favorable phenotypic alterations in trophoblasts isolated from preeclamptic placentas change to control values when cultivated *in vitro*.^80^ The authors concluded that the adverse trophoblast phenotype noticed in preeclamptic patients is at least partly induced by the uterine environment, implicating that the pathological phenotype of trophoblasts noticed in PE could be reversed by therapeutically targeting the uterine environment.

In the light of these data, it would be highly interesting to study potential phenotypic alterations in decidual cell populations obtained from complicated pregnancies and, if present, to test their influence on EVT function.

## Conclusion

The study of human trophoblast subtype-specific function and their control is challenging since there are various limitations imposed by ethical considerations and the high diversity in placenta development among animal species. Although our knowledge, in particular about early events during trophoblast invasion, remains scarce recent advances and increasing interest in the field promise progress in the near future. In this context, some interesting questions yet to be answered include: • Can imCTBs breach the basal membrane of spiral arteries and thus contributing to intramural remodeling of these vessels? • Do iCTBs and/or imCTBs invade and remodel decidual veins? • Do endometrial glands contribute to early placental development and is iCTB-mediated invasion and replacement of the glandular epithelium of functional relevance? • To which extent does proper EVT function depend on paracrine signals from the decidua? • Are complicated pregnancies with impaired EVT function also associated with adverse decidual cell phenotypes? In summary, further research is necessary to receive a clear picture of EVT subtype-specific functions and in understanding the dynamics in the interplay between trophoblasts and decidual cells (Fig. 1).

## Abbreviations

ADAM12: a disintegrin and metalloproteinase 12
DSC: decidual stromal cell
ECM: extracellular matrix
eCTB: endothelial cytotrophoblast
EGF: epidermal growth factor
EGFR: epidermal growth factor receptor
EVT: extravillous trophoblast
hCG: human chorionic gonadotropin
HLA: human leukocyte antigen
hPL: human placental lactogen
ICM: inner cell mass
iCTB: interstitial cytotrophoblast
imCTB: intramural cytotrophoblast
ITGA: integrin α
IUGR: intrauterine growth restriction
MMP: matrixmetalloproteinase
PE: preeclampsia
SRSF1: serine/arginine-rich protein serine/arginine-rich splicing factor 1
ST: syncytiotrophoblast
TCF4: t-cell factor 4
TE: trophectoderm
TGFβ3: tumor growth factor β3
uNK: uterine natural killer cell
vCTB: villous cytotrophoblast.
